# The Study of Intelligent Vehicle Navigation Path Based on Behavior Coordination of Particle Swarm

**DOI:** 10.1155/2016/6540807

**Published:** 2016-01-12

**Authors:** Gaining Han, Weiping Fu, Wen Wang

**Affiliations:** ^1^School of Mechanical and Precision Instrument Engineering, Xi'an University of Technology, Xi'an, Shaanxi 710048, China; ^2^Information Engineering Department, Xianyang Normal University, Xianyang, Shaanxi 712000, China

## Abstract

In the behavior dynamics model, behavior competition leads to the shock problem of the intelligent vehicle navigation path, because of the simultaneous occurrence of the time-variant target behavior and obstacle avoidance behavior. Considering the safety and real-time of intelligent vehicle, the particle swarm optimization (PSO) algorithm is proposed to solve these problems for the optimization of weight coefficients of the heading angle and the path velocity. Firstly, according to the behavior dynamics model, the fitness function is defined concerning the intelligent vehicle driving characteristics, the distance between intelligent vehicle and obstacle, and distance of intelligent vehicle and target. Secondly, behavior coordination parameters that minimize the fitness function are obtained by particle swarm optimization algorithms. Finally, the simulation results show that the optimization method and its fitness function can improve the perturbations of the vehicle planning path and real-time and reliability.

## 1. Introduction

In recent years, with the increase in the number of cars, traffic accidents happen more and more frequently, most of which are caused by man-made mistakes and serious threat to people's live and social stability. Thus, “car-people-road” mode is converted to “car-computer-road” mode, which can be liberated from driving environment and is one of the effective ways to reduce traffic accident. The unmanned ground vehicle has become a hot issue domestic and international research [1–3]. The DARPA (Defense Advanced Research Projects Agency) proposed a series of control technology for the unmanned vehicle and digital road traffic control scheme under the urban road environment, which is the most important for the path planning and controlling [4–9].

This paper mainly focused on the intelligent vehicle navigation problems, especially the path planning, and guiding the vehicles to the target position. At present, the traditional path planning methods, including potential field and grid method, are prone to local minimum problems [10, 11]. The bionic path planning algorithms, including genetic algorithm, ant colony algorithm, and particle swarm algorithm, need to determine the coding scheme and the optimization of objective function [12–17]. The intelligent path planning algorithms, including BP neural network, ANN, and fuzzy neural network learning methods, need constant learning and training to obtain the optimal path; the network settings of the intermediate nodes of these methods are often dependent on experience and constant trial and error [18–21]. These are only from the perspective of the planning. However, the navigation path of the intelligent vehicle should not only contain trace paths but also contain the time information (namely, speed), for real-time tracking control. The above methods including field method, bionic method, and intelligent information do not include the time in the path planning.

In 1995, behavioral dynamics method is first proposed by Schöner et al. [22]; it has been applied to a mobile robot system, complex structured environment navigation control, machine control arms, and multirobot formations navigation control [23–31]. Due to its outstanding performance, scholars began to pay attention to relevant theorem studying and application research [32–38]. Behavior dynamics method is introduced into the intelligent vehicle navigation planning and in-depth research and experiments verify the feasibility of this method.

The aim of the paper is to discuss the intelligent vehicle path planning problem in dynamic environment. In the urban environment, the road environment information is acquired through the vehicle sensor, which is indispensable for path planning and obstacles avoiding. Behavior dynamics method is used for the path planning to generate competition behavior problem, while the particle swarm optimization algorithm is adopted to improve the behavior coordination.

The rest of the paper is as follows: Section 2 introduces behavior dynamics method. The behavior coordination of the standard particle swarm optimization algorithm is introduced in Section 3. Simulation results of illustration examples and discussion are presented in Section 4. Finally, in Section 5, the conclusion and further discussion are given.

## 2. Behavior Dynamics

The behavior dynamics were included towards the goal of behavior and obstacle avoidance behavior. Using the behavior character of behavior dynamics plan according to the local target from a starting point of a path (namely, the intelligent vehicle navigation path) and satisfying the vehicle in the driveway can avoid the pavement and all sorts of obstacles to the dynamic environment. The heading angle and the path velocity in behavior dynamics model could be represented as the following form [26]:

(1) ψ˙=f1ψ,P,v˙=f2v,P,

where *P* is position of the intelligent vehicle in the world coordinator system and the parameters are the heading angle *ψ* and the path velocity *v* of the intelligent vehicle, which generally act as the behavioral variables. $\dot{\psi }$ and $\dot{v}$ define the rate of the heading angle and velocity as a function of their current values.

### 2.1. Behavior Dynamics Modeling

**Figure 1:**
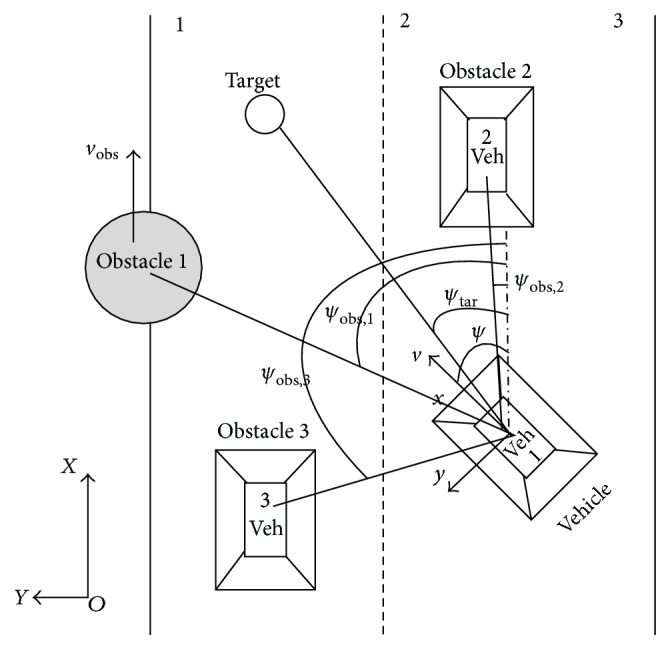
Variables represented in the dynamics.

The dynamic approach to path generation of the intelligent vehicles employs *ψ* and *v* as the planning variable [22, 23]. The path planning is continuous in a time course of *ψ* and *v*; the target directions *ψ*_tar_ relative to the world coordinate system in which target lie from the view of the intelligent vehicle and the obstacle directions *ψ*_obs,*i*_ in which obstacles lie from the viewpoint of the vehicle relative to the world coordinate system, *ψ*_tar_ and *ψ*_obs,*i*_, are represented by attractive and repulsive force-lets acting on heading direction (Figure 1). The velocity of target *v*_tar_ and the velocity of obstacle *v*_obs_ are constraints that are represented by attractive and repulsive force-lets acting on the path velocity. In the dynamics system, the variables are the direction angle and the path velocity lying at obstacles and target from the current position of the intelligent vehicle shown in Figure 1.

According to the behavior variables and the behavior pattern of the intelligent vehicle, the target and obstacle avoidance of dynamics model is built.

#### 2.1.1. The Target of Behavioral Models

Toward target behavior is the behavior of the intelligent vehicle to reach its destination. The dynamic behavior of the attractor equation is expected with negative slope and asymptotic speed converges to a stable fixed point. The attractors of the equation defined the behavioral variables so that the system is ensured to be at all times in a stable state. The heading angle and the path velocity of the behavior model are established as the following.

*(1) Heading Direction Behavior Dynamics Model.* In the behavior dynamics model, the heading angle is specified as a time-variant term at any time *t*. Ψ is an angle and is cyclic and defined as *ψ* ∈ [−*π*/2, *π*/2] in the world coordinates, and the *x*-axis is always consistent with the direction of the road. In [24] the heading direction of behavior dynamics for target acquisition can be defined by

(2) ψ˙=ftarψ=−λtartan⁡ψ−ψtar,ψtar=arctan⁡Ptary−PvehyPtarx−Pvehx,

where *λ*_tar_ > 0 sets the attraction strength of the vehicle's heading variable, *ψ*_tar_ is the angle between the target and the intelligent vehicle in world coordinate system, and *ψ*_tar_ is the attractor in dynamics system. *f*_tar_(*ψ*) is an attractive force-let and converges to the value so that *ψ* goes towards the intended target-state. *P*_tar_(*P*_tar_ *x*, *P*_tar_ *y*) is target position in the world coordinator system and is the time-variant target, and *P*_veh_(*P*_veh_ *x*, *P*_veh_ *y*) is intelligent vehicle position in the world coordinator system.

*(2) The Path Velocity Behavior Dynamics Model*. Besides heading direction, in order to ensure the formation and maintenance of the stabilization, path velocity must be adjusted real-time. In terms of path velocity, considering that the security and stability of the vehicle system must meet with safety distance from maximum contact time *T*_max_ = (*d*_tar_ − *D*_*s*_)/*v*, *D*_*s*_ is a minimum safe following distance and *d*_tar_ is the distance to the target. *v*_tar_ is an attractor of velocity, and *v* goes towards the required velocity *v*_tar_. Literature [26, 34, 35] used linear velocity equation, with certain constraints. In this study, [25] is used to define the velocity behavior dynamics the equation towards the target:

(3) $$\begin{array}{c} \dot{v}=f_{tar,v}\left(\;v\;\right)=-\lambda_{tar,v}\left(\;v-v_{tar}\;\right)\exp\left[\;\frac{{\left(v-v_{tar}\right)}^{2}}{2\sigma_{v}^{2}}\;\right], \end{array}$$

where *λ*_tar,*v*_ is strength factor, *v*_tar_ is the desired vehicle velocity, and *σ*_*v*_ is scope attractors calculated in [36].

#### 2.1.2. Obstacle Avoidance Behavior Model

Obstacle avoidance behavior can be divided into heading direction and velocity behavior.

*(1) Heading Direction Behavior Dynamics Model*. When the intelligent vehicle is driving in the way of the target road, it is time to avoid the static or moving obstacles on the road to reach the destination safely. *ψ*_obs,*i*_ is called the repeller, which is unstable point *ψ*_obs,*i*_ to make the influence of an obstacle go to zero in behavioral dynamics system; *ψ*_obs,*i*_ defined the behavioral variables. Reference [24] sets the heading direction in the obstacle avoiding behavior using the following equation:

(4) ψ˙obs,i=fobs,iψobs,i=λobs,iψ−ψobs,iexp⁡−Cdobs,i·exp⁡−ψ−ψobs,i22σi2,

where *λ*_obs,*i*_ is the repulsion strength, *d*_obs,*i*_ is the distance between the obstacles and intelligent vehicle, which can continuously be estimated using sensory inputs, *C* is the repulsive force of attenuation coefficient with the increase of distance, *σ*_*i*_ is the angular scope of the repeller, and *σ*_*v*_ is velocity scope of the repeller [36]. This implies that the strength and angular range of the repulsion become stronger as the intelligent vehicle gets closer to obstacles.

Multiple obstacles heading direction of behavior dynamics equation can be written as

(5) $$\begin{array}{c} {\dot{\psi }}_{obs}=F_{obs}=\sum_{i}f_{obs,i}\left(\;\psi_{obs,i}\;\right). \end{array}$$

*(2) The Path Velocity Behavior Dynamics Model*. The velocity of the intelligent vehicle is related to the distance between vehicles and obstacles *d*_obs_ and also to the safety distance *D*_*s*_. Similar to (3), the path velocity could be modeled as

(6) $$\begin{array}{c} \dot{v}=f_{obs,i}\left(\;v\;\right)=-\lambda_{obs,v}\left(\;v-v_{obs,i}\;\right)\exp\left[\;\frac{{\left(v-v_{obs,i}\right)}^{2}}{2\sigma_{v}^{2}}\;\right], \end{array}$$

where *λ*_obs,*v*_ is velocity to the repeller of velocity intensity factor, the repeller can be changed by adjusting *λ*_obs,*v*_, *v*_obs,*i*_ is the required obstacle velocity, and *σ*_*v*_ is scope the repeller calculated in [36].

### 2.2. Behavior Dynamics Coordination

After the establishment of the various dynamics model, navigation planning demands the fusion of several behavior variants; in practical applications, it is necessary to fuse the behavior various and conduct planning for the vehicle. Consider a fusion of two actions:

(7) ψ˙=ωobsFobs+ωtarftar,

(8) v˙=λobsfobs,iv+λtarftarv,

where *ω*_obs_, *ω*_tar_, *λ*_obs_, *λ*_tar_ are the weight coefficients and behavior of fusion is not considered in literature [36], where four weight coefficients are equal to 1. Thereof, when the target behavior and obstacle avoidance behavior occur simultaneously, two types of behavior lead to a perturbation of the intelligent vehicle. Under the absence of behavior of fusion, the target behavior and obstacle avoidance behavior occur simultaneously, which leads to the perturbation of the vehicle. In order to solve this problem, behavior coordination was studied in depth.

*(1) Heading Direction Behavior Fusion*. From (7), in the process of driving, it can be seen that there only exists the target behavior with no obstacles in the environment, so *ω*_obs_ = 0, *ω*_tar_ = 1. When obstacles exist in the process of intelligent vehicle driving, obstacle avoidance behavior is stronger than toward the target behavior, so parameters *ω*_obs_ = 1, *ω*_tar_ = 0. When the intelligent vehicle passes through obstacles, the main obstacle avoidance behavior turned toward the target behavior. Similarly, when the vehicle encounters obstacles, the vehicle behavior turns from “toward target” to “obstacle avoidance” along the direction of gradient between the two states, associating with the increasing weight of the obstacle avoidance behavior. Therefore, the vehicle direction perturbations problem arises with the inharmoniousness behavior switching because of the rough coordination.

*(2) The Path Velocity Behavior Fusion*. Similarly, from (8), when there are no obstacles or the obstacles are static, there is only velocity towards the target behavior: the parameters *λ*_obs_ = 0, *λ*_tar_ = 1. When obstacles are in motion in the process of intelligent vehicle driving, obstacle avoidance behavior is stronger than toward target behavior, the parameters *λ*_obs_ = 1, *λ*_tar_ = 0 in (8). When the vehicle is away from obstacles, the velocity of behavior is directed towards the target. Due to the change of behavior, speed behavior alternates from moving towards the target to obstacle avoidance, so that the two behaviors can smooth transition, which will depend on two behaviors of coordination. Otherwise there will be fast or slow phenomenon for the velocity of intelligent vehicle.

## 3. Particle Swarm Optimization Algorithm

According to many research literatures [32, 33, 35, 39], behavior of competitive dynamics method is used for behavior coordination. Besides the differential form of dynamics equation, the competition dynamics model is still the differential equation and relates to the stability of the system making behavior coordination more complicated. In the literature [38], heading direction angle coefficient of the mobile robot is optimized by genetic algorithm, with the velocity left constant. Reference [39] using particle swarm optimization algorithm, mainly for the interaction between multiple mobile robots for the angle of coordinate, did not consider speed. In this paper, it introduced the behavior from the direction angle and linear velocity at the same time by the particle swarm optimization algorithm to strengthen the problem of the intelligent vehicle navigation path.

### 3.1. Particle Swarm Optimization Algorithm

The particle swarm optimization algorithm has many advantages such as being easy to describe, adjusting less parameters, and having fast converging speed, so that it has become a hotspot in intelligent optimization and evolution computing once proposed. It has been widely used in the function optimization, dynamic environment optimization, neural network training, and fuzzy system control applications [40].

In PSO, each potential solution to optimization problem is a bird in the search space, called a particle. All particles have a function that is optimized by the decision on the fitness value. Each particle has a speed that decided they fly out of direction and distance, and the optimal particles are to follow the current search in the solution space. Optimization starts to initialize a set of random particles (random solutions) and determines the merits of the solution by the fitness function. Each particle iterative process contains two extremes: the individual extreme and global extreme; individual extreme cognitive level represents particles themselves, and the global extremes represent the social cognitive level; the final optimal solution is obtained after several iterations [40].

The goal of optimizing function search space for *m*-dimensional (namely, the number of optimization variables) is set, the number of particles is *n*, the *i* particle position is *X*_*i*_ = (*X*_*i*1_, *X*_*i*2_,…, *X*_*im*_)^*T*^, and speed is *V*_*i*_ = (*V*_*i*1_, *V*_*i*2_,…, *V*_*im*_)^*T*^. The course of the flight in accordance with (7) and (8) to update their own position and speed of the particle. *P*_*i*_ = (*P*_*i*1_, *P*_*i*2_,…, *X*_*im*_)^*T*^ for the individual pbest; *P*_*g*_ = (*P*_*g*1_, *P*_*g*2_,…, *X*_*gm*_)^*T*^ for the global gbest:

(9) Vikt+1=wVikt+c1r1Pikt−Xikt+c2r2Pgkt−Xikt,Xikt+1=Xikt+Vikt+1,

where *k* = 1,2,…, *m*, *i* = 1,2,…, *n*, *t* is the number of iterations, *w* is weight coefficient for the particle velocity, and the greater the value of *w*, the better global searching ability for PSO. *r*_1_, *r*_2_ are a random number in [0,1], and *c*_1_, *c*_2_ are limiting factors for acceleration. In iterative process, the particle swarm optimization algorithm has no specific mechanisms to control the velocity of the particles; the speed of the particles to limit the particle speed of each dimension is within [*V*_min_, *V*_max_]; the position of each dimension is limited within [*X*_min_, *X*_max_].

### 3.2. PSO Implementation of Behavior Coordination

Based on the driving characteristics of the intelligent vehicle, at a certain velocity, when the behavior toward the target and obstacle avoidance behavior occurs alternately to reach the destination safely, it becomes a major obstacle avoidance behavior, obstacle avoidance behavior becomes a major behavior and towards the target of behavior will be weakened. When passing through the obstacles, toward the target, behavior becomes dominant. Behavior dynamics analysis through the front, towards the target, and obstacle avoidance behavior including heading direction and the path velocity behavior, the weight coefficient of heading direction, and the path velocity are optimized by particle swarm optimization algorithm, thereby eliminating perturbations; the intelligent vehicle can smoothly move through obstacles to reach the target location.

In this paper, employing the method of PSO, optimization variables are expressed by the particle; the particles set is *X*_*ψ*_ = (*ω*_obs_, *ω*_tar_) and *X*_*v*_ = (*λ*_obs_, *λ*_tar_). The fitness function reflects the relationship between the intelligent vehicle and the environment. When the obstacle is very close to the intelligent vehicle, the avoidance obstacle behavior plays the leading factor. When the intelligent vehicle is away from object, the target of behavior plays a decisive role; the fitness function is designed as follows:

(10) $$\begin{array}{c} F_{\psi }=\left|\;\frac{d_{obs}/\left(\psi -\psi_{tar}\right)}{d_{tar}/\left(\psi -\psi_{obs}\right)}\;\right|. \end{array}$$

As proposed in [41], (7) and (8) could be solved by the fourth-order Runge-Kutta method. Besides, *ω*_obs_, *ω*_tar_ are confirmed by the extreme value of fitness function using the particle swarm optimization algorithm.

Similarly, the velocity of behavior of coordination fitness function is

(11) $$\begin{array}{c} F_{v}=\left|\;\frac{d_{obs}\left(v-v_{tar}\right)}{d_{tar}\left(v-v_{obs}\right)}\;\right|. \end{array}$$

Using the particle swarm optimization algorithm for its function extreme value, *λ*_obs_, *λ*_tar_ can be obtained.

## 4. The Simulation Analysis

In order to verify the behavior dynamics method as well as behavior coordination algorithm is feasible in the intelligent vehicle path navigation, we carried out the experiment under the condition of the different pavement environment and avoiding obstacle, security requirements like lane keeping, vehicle following, lane changing, and overtaking. Follow these steps to carry out experiment:

1. Establish the system model with unknown parameters of the intelligent vehicle, the sensors, and the road environment. These parameters are all detected by vehicular sensors and used to parameterize corresponding term such as road environment, starting point and target point of the driving, initial velocity and heading direction of the vehicle, position of the obstacles, detecting range and distance of the sensor, and so forth.
2. During the process of driving, obstacles beyond detection scope are according to the behavior dynamics model towards the target for navigation path. When obstacles are detected, there is a use of behavior dynamics of the obstacle avoidance behavior and towards the target behavior of the coordination algorithm for navigation path. The intelligent vehicle in driving, when detecting obstacles outside the safe distance, is according to the dynamics of behavior towards the goal of behavior for navigation path, when detecting obstacles within the safe distance, according to the behavior of dynamic obstacle avoidance behavior and towards the target behavior of the coordination for navigation path.
3. Nonstationary conditions of the vehicle continuously determine the current vehicle position and the target position; stop the iteration after reaching the target; otherwise, repeat (2).

In this paper, MATLAB R2013a simulation experiments, as shown in Figure 1. the simulation parameters: intelligent vehicle initialization parameters for VehiclePos = [21,3, 90,3.6,8, 90,1] (initial *x*, *y* coordinates, heading, speed, distance perception, perception range, vehicle size), reference vehicles initialization parameters for VehicleRunPos1 = [20,15,90,4.3,120,1], the target position initialization parameters for TargetPos = [VehicleRunPos1 (2), VehicleRunPos1 (2)](target *x* and *y* coordinates), the obstacle position initialization parameters for ObstaclePos = [12  5  90  2  2; 27  30–60  2  1.5] (*x* and *y* coordinates of the obstacle heading direction, speed, size of obstacles), perceive the sector range [−30°300°], scene size of [50  50]; Particle swarm optimization parameters *c*1 = *c*2 = 2, *w* = 0.8, *r*1 = *r*2 = rand⁡(1), *t* = 20, *m* = 2, *n* = 20, [*V*_min_, *V*_max_] = [0,1], [*X*_min_, *X*_max_] = [0,1].

In figure, blue filled circles are an obstacle, red filled circle is detected in the movement of obstacles, black thick line and blue dotted line are for the lane, green rectangle is for intelligent vehicle, blue sector is for intelligent vehicle sensing area, black rectangles are for the target vehicle, and red signs are for intelligent vehicle running track.

**Figure 2:**
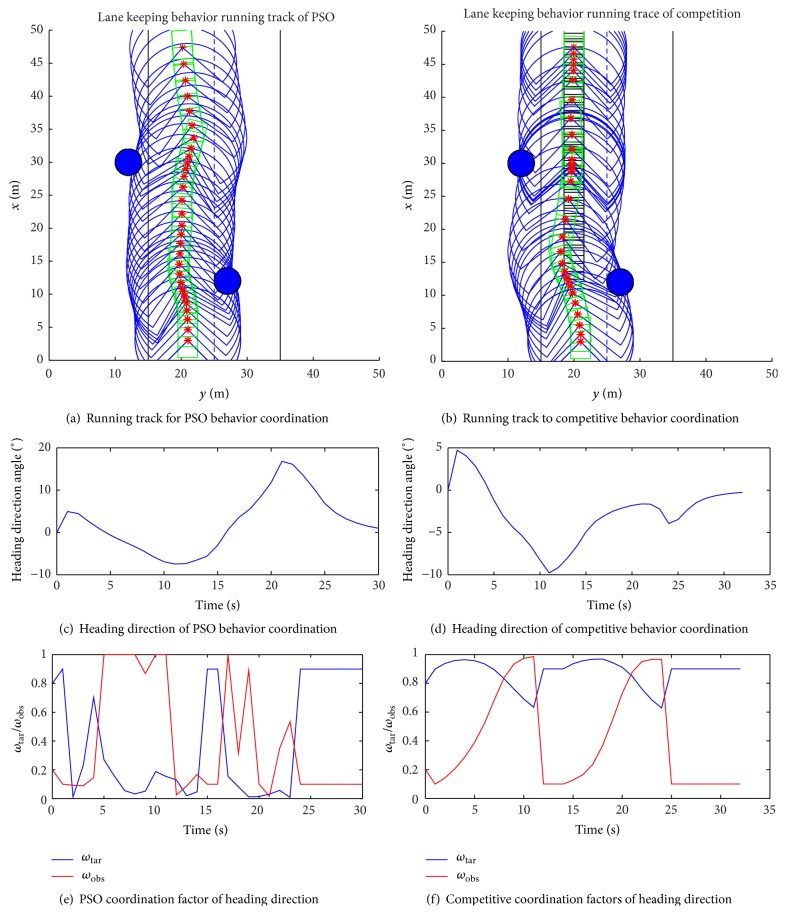
Straight to heading direction coordinate.

The simulation results are shown in Figure 2. In the same scene, two kinds of behavior coordination algorithm optimize the simulation results shown in Figure 2, so the velocity of obstacles behavior weight coefficient is 0, only weight coefficient of heading direction behavior coordination.  Figure 2(a) shows running track for PSO behavior coordination in the lane for safe obstacle avoidance and the destination; Figure 2(b) shows running track to Competitive behavior coordination for the lane safe obstacle avoidance and the destination. Figures 2(c) and 2(d) show the intelligent vehicle heading direction variation for PSO behavior coordination and competition behavior coordination. Figures 2(e) and 2(f) show the weight coefficient variation in heading direction for PSO behavior coordination and competition behavior coordination. From the simulation data and diagram it can be seen as in PSO behavior to coordinate the navigation path and heading direction change closer to the actual vehicle characteristics of the vehicle, while the angle change is too large for competitive behavior coordinated. For Figures 2(e) and 2(f) the heading direction of obstacle avoidance and towards the target weight coefficient optimization and the particle swarm optimization method of two parameters optimization more conform to the behavior of the competition law; the particle swarm optimization method is easy to realize in the design, less parameter adjustment, fast convergence, and so on. However, using the method of the differential equation of competition, more parameters are adjusted, making the coordination more complicated.

**Figure 3:**
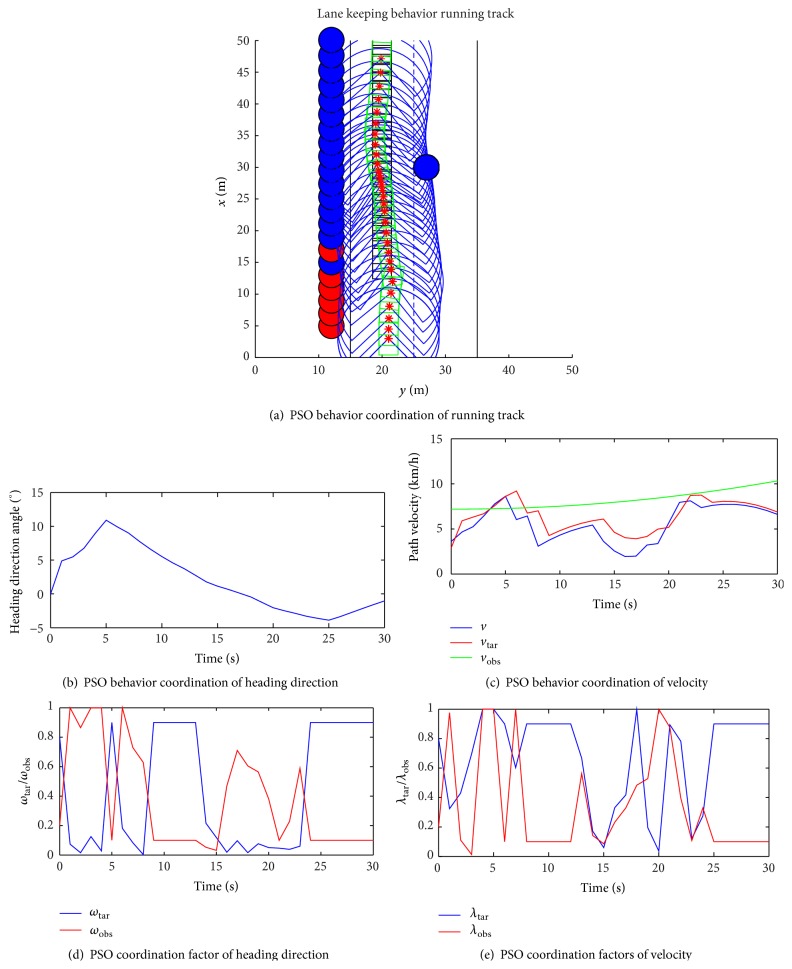
Straight to heading direction and velocity of PSO behavior coordination.

Figure 3 is the optimization effect of the PSO heading direction and velocity behavior coordination in dynamic obstacles.  Figure 3(a) shows PSO behavior coordination to the navigation path of intelligent vehicle, Figures 3(b) and 3(c) show PSO behavior coordination in the heading direction and velocity changes, and Figures 3(d) and 3(e) show the heading direction and velocity of weight coefficient of variation to PSO behavior coordination. Figures 3(a) and 3(b) can be seen as the simulation trajectory and angle change is consistent with the actual vehicle. Figures 3(d) and 3(e) can be seen as heading direction and velocity weight coefficient optimization point of view; PSO weight coefficient is more consistent with the competition behavior change law and can control the intelligent vehicle safe avoidance obstacle, towards the target.

**Figure 4:**
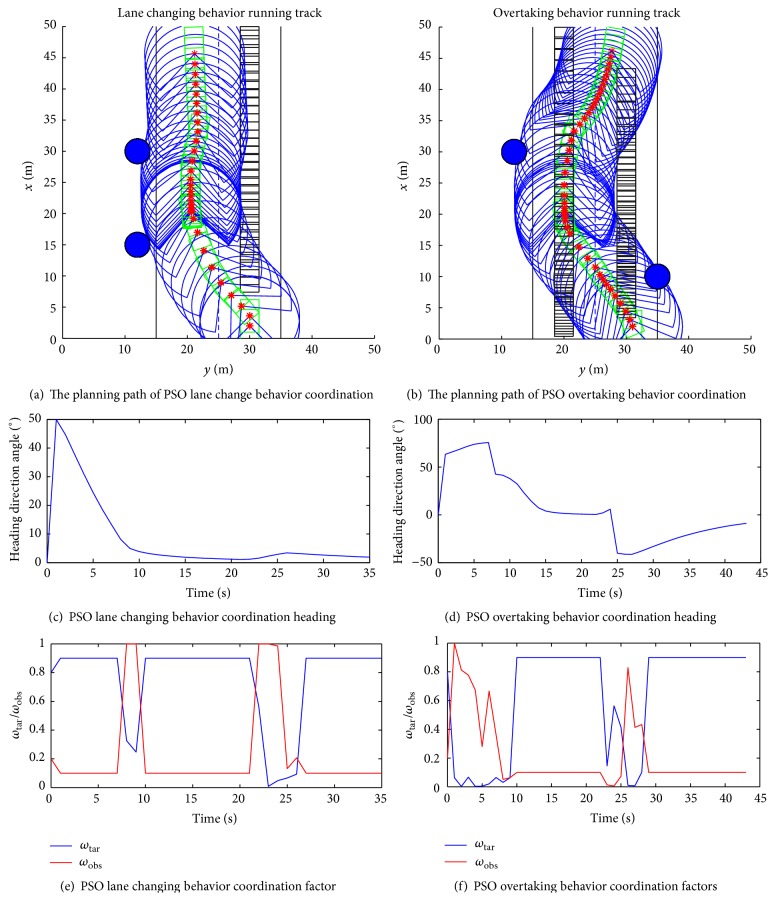
Lane changing and overtaking heading direction coordination behavior.

Figure 4 shows lane changing and overtaking using behavior dynamics method and PSO behavior coordination, from running track of the intelligent vehicle, heading direction change, and behavioral weight factors variation law can be seen; behavior dynamics can be well for path planning of the intelligent vehicle.

Simulation results show that behavior dynamics method and PSO behavior coordination can effectively navigate path of the intelligent vehicle for the vehicle follow, lane keeping, lane changing, and overtaking behavior. PSO algorithm solves the obstacle avoidance behavior toward the target behavior coordination due to behavior competition arising perturbations. In the process of driving, at the same time, the change rule of heading direction and driving direction are consistent, and the vehicle's velocity is adjusted in the process of obstacle avoidance to remain consistent with the actual traffic characteristics.

## 5. Conclusion

(1) Based on the characteristics of intelligent vehicle driving, combined with behavioral dynamics method, we established method of the navigation path of the intelligent vehicle. The target is the time-variant.

(2) Using PSO algorithm and competition behavior algorithm toward the target and avoidance weight coefficients to optimize shows that PSO algorithm in behavior weight coefficient optimization has advantages: fast convergence and less parameter setting, using behavioral dynamics method to realize the control of the intelligent vehicle for the lateral heading control and the longitudinal velocity control, so that the intelligent vehicle along a desired direction path can pass safely through the obstacles to target position.

In addition, using the behavior dynamics to the navigation path can provide the relevant parameters for the next motion control. Lateral control is concerned with steering the vehicle automatically to follow the reference path. The longitudinal control is concerned with accelerating the vehicle automatically. The variable behavior dynamics is the relation between $\dot{\psi }=f_{tar}$ and the yaw, the relation of $\dot{v}=f_{tar,v}(v)$ longitudinal acceleration. The intelligent vehicle is controlled by using actuators such as the brakes, the accelerator, and the steering wheel so that it adheres to the reference path [42].

(3) The experimental results show that behavioral dynamics method has the real-time performance and reliability, and the PSO algorithm can be a good alternative to the behavior coordination problems.
